# We are what we eat: Regulatory gaps in the United States that put our health at risk

**DOI:** 10.1371/journal.pbio.2003578

**Published:** 2017-12-20

**Authors:** Maricel V. Maffini, Thomas G. Neltner, Sarah Vogel

**Affiliations:** 1 Independent consultant, Germantown, Maryland, United States of America; 2 Environmental Defense Fund, Washington DC, United States of America; National Institute of Environmental Health Sciences, United States of America

## Abstract

The American diet has changed dramatically since 1958, when Congress gave the United States Food and Drug Administration (FDA) the authority to ensure the safety of chemicals in food. Since then, thousands of chemicals have entered the food system. Yet their long-term, chronic effects have been woefully understudied, their health risks inadequately assessed. The FDA has been sluggish in considering scientific knowledge about the impact of exposures—particularly at low levels and during susceptible developmental stages. The agency’s failure to adequately account for the risks of perchlorate—a well-characterized endocrine-disrupting chemical—to vulnerable populations is representative of systemic problems plaguing the regulation of chemicals in food. Today, we are faced with a regulatory system that, weakened by decades of limited resources, has fallen short of fully enforcing its mandates. The FDA’s inability to effectively manage the safety of hundreds of chemicals is putting our children’s health at risk.

This Perspective is part of the *Challenges in Environmental Health: Closing the Gap between Evidence and Regulations Collection*.

## Introduction

Over the course of the 1950s, Congress debated legislation that sought to address rising concerns about the safety of hundreds of new, industrially produced chemicals transforming the way Americans grew, packaged, processed, and transported the food they ate. In 1958, the Food Additive Amendment (FAA) to the 1938 Federal Food, Drug and Cosmetics Act (FFDCA) [1] was signed into law, providing new authority to the Food and Drug Administration (FDA) to ensure the safety of chemicals added to the food supply. [2] Among other considerations, Congress intended that the FDA address the chronic and cumulative exposures to chemicals in the food supply when considering safety. [3] The American diet, just like that of most developed countries, has changed dramatically in the nearly 60 years since the law passed. Today, there are more than 10,000 chemicals [4]—commonly referred to as food additives—allowed in food, which presents a critical challenge to the FDA’s ability to effectively assess and manage the safety of all of these chemicals. This challenge has become particularly evident in the face of mounting scientific evidence that some of these chemicals—including endocrine-disrupting agents—can interact with biological systems at exceedingly low, chronic levels of exposure and result in adverse health impacts, especially when exposures occur during pregnancy or early childhood [5].

The ongoing presence of various chemicals in the food supply that have been associated with significant health risks [6,7] indicates that the FDA’s scientific decision-making processes are inadequate to take into account the best available evidence and methods for assessing the chronic and cumulative effects of chemicals. In 1982, a select committee of experts provided the FDA with recommendations to improve chemical safety. Among their main issues were to pay special attention to the effects of chemicals on behavioral changes and the endocrine system; not to assume that chemicals below a certain concentration are not hazardous; and to ensure susceptible populations were protected [8]. Unfortunately, the FDA has not incorporated the great majority of these recommendations made over three decades ago [9]. As a result, the health of children and vulnerable populations are particularly at risk.

There are a number of interrelated factors that contribute to creating a gap between the intent of the law regulating chemicals in food and the agency’s practices; these include limited agency funding that reduces available resources for updating scientific practices and guidance and the fact that an absence of internal resources generates a dependency on the regulated industry, including its implementation and interpretation of the law. Importantly, the interpretation of the law to allow companies to make safety determinations for “generally recognized as safe” (GRAS) substances without notifying the FDA has tied the agency’s hands, making it difficult to obtain information about a chemical’s safety and generating a strong disincentive to update agency science.

Adequately addressing this gap between intent and practice demands more than just a dedication to science; it also will require a renewed commitment to the agency itself through resources that can bring new talent and ideas to the agency and the authority to get the information it needs, such as reporting on new uses [10]. At a time when federal agencies in charge of protecting public health face potential budget cuts and political leaders undermine the very practice of scientific inquiry, many health safeguards we take too easily for granted are at stake [11]. Among them are the safety of the chemicals in the food we eat every day.

## A case study: Perchlorate

The chemical perchlorate illustrates an expanding disconnect between the original intent of the 1958 food law as it relates to managing chronic health impacts of chemicals and the agency’s practice for allowing chemicals into the food supply. Because the chemical’s mode of action is well understood and undisputed—inhibition of the transport of iodine from the blood to the thyroid gland—it also makes for a compelling example. Perchlorate is a naturally occurring and man-made chemical that quickly dissolves in water and organic solvents and persists in the environment [12]. It has been found in the urine of all Americans tested [13]. While it leaves the body quickly, perchlorate persists in the environment for many years. The risks of chronic exposure to perchlorate are well described, as is the evidence of its widespread presence in the environment [14,15,12]. The FDA has approved perchlorate’s use as a food contact substance twice. The first time was in 1963 for its use in sealing gaskets for food containers; and then, in 2005, it was approved for use as a conductivity enhancer or antistatic agent in dry food packaging [16].

Perchlorate is perhaps most commonly known for its uses in rocket fuel, explosives, and fireworks and as a contaminant of nitrate-based Chilean fertilizer [12]. Perchlorate enters the body through food and water, with food being the main contributor [17]; perchlorate contaminates food through two primary uses: as an antistatic agent in any plastic material that contacts dry food, including final and bulk packaging, and as a contaminant of hypochlorite bleach, which oxidizes to form chlorate and then perchlorate with time and inadequate management [18]. Bleach—a pesticide—is widely used to sanitize food surfaces in food-manufacturing and -processing facilities; it is also a food additive approved by the FDA to wash and peel fruits and vegetables [19].

Perchlorate primarily affects the normal functioning of the thyroid gland by inhibiting the transport of iodine from the blood into the organ. Iodine is an essential element needed to produce thyroid hormone—which plays an important role in controlling metabolism and is critical in regulating fetal and infant brain development. Because perchlorate is such a strong inhibitor of iodine transport [20], pregnant women, infants, and children with inadequate iodine consumption are the most vulnerable, and exposure to the chemical greatly increases the risk of impaired neurodevelopment [14].

At least 20% of pregnant American women consume so little iodine that any exposure to perchlorate presents a significant risk of adverse neurological development in the fetus [21]. Effects caused by lowering thyroid hormone levels during brain development are likely to be subtle and may be manifested as intellectual deficiencies and other chronic developmental delays. Emerging scientific evidence demonstrates that perchlorate exposure decreases thyroid hormone levels during pregnancy [14] and that children born to mothers with borderline thyroid deficiency and who were exposed to perchlorate during the first trimester had decreased intelligence quotient (IQ) [15].

It is very likely that the FDA’s first approval of perchlorate use in contact with food more than 50 years ago was based on limited or no safety data. However, available scientific evidence on perchlorate targeting the thyroid gland and contemporary controversies about safe levels of perchlorate exposure from drinking water [22] make its 2005 approval [23] puzzling. Over the past decade, the evidence for the adverse neurological effects of low-level exposure to perchlorate has become stronger, yet the agency continues to stand behind its approval of perchlorate’s use in dry food packaging and food handling equipment [16].

## Outdated scientific methods built on flawed assumptions led to bad decisions

It’s worth then exploring how the FDA considers perchlorate to be safe for use in food. A fundamental, long-standing assumption informing the agency’s determination of safe uses is that, unless the chemical is a carcinogen, there is a threshold effect. That is, below a certain level of exposure, there is no adverse effect. The FDA assumed that perchlorate was safe at low doses. In 1982, a committee of experts told the agency the assumption of a threshold effect was “scientifically untenable” and ignored the possibility of irreversible organ function alteration [9]. And yet, in 1995, the FDA responded by issuing a Threshold of Regulation rule [24] that codified exemptions of chemicals from regulation as food additives if the amount stayed below a predetermined level in the diet of 0.5 parts per billion without a rigorous evaluation of its safety.

The second reason for allowing endocrine disruptors such as perchlorate can be found in the FDA’s guidance for chemicals used in packaging or food-handling equipment. The guidance is based on tiers of exposure below which limited (e.g., gene toxicity) or no safety data are expected to be generated [25]. The FDA has also fallen short in its efforts to update its scientific practices. A case in point is the 1982 publication of the Redbook, the agency’s guidance for the food industry on chemical testing methods [26]. Although it was reviewed in the 1990s and 2000 [27], none of the major scientific advances in neurodevelopment, endocrinology, reproductive biology, and immunology were reflected in the guidance. For example, unlike the Organization for Economic Cooperation and Development [28], the FDA does not have guidelines for testing developmental neurotoxicity [27]. In 2014, the FDA announced it was taking “steps to strengthen its program to assess the safety of chemicals in food,” which includes another revision of the Redbook [29].

Importantly, the agency lacks guidance on the best practice for evaluating chronic diseases caused by a single chemical or multiple chemicals. The result is that there are no testing requirements to demonstrate the effects of very low or cumulative exposures that occur in the diet. The agency’s decision on perchlorate under the Threshold of Regulation rule did not consider the cumulative effect of perchlorate, nitrates, and thiocyanate. These latter two chemicals are also present in the diet and, like perchlorate, affect the thyroid gland by inhibiting the transport of iodine, although they are weaker inhibitors compared to perchlorate [20]. This is a clear-cut example of what Congress intended when it mandated the FDA to assess the cumulative effects of co-exposures and investigate their potential chronic health effects.

In sum, the FDA has fallen short in its responsibility to identify and thoroughly evaluate what chemicals may cause the type of long-lasting diseases that have become so prevalent, including behavioral and neurodevelopmental disorders associated with perchlorate exposure. In considering the safety of perchlorate, the agency assumed a threshold effect and did not require data on the neurological effects of low-dose, chronic exposures nor consider the impact of cumulative effects of other chemicals also affecting the thyroid gland. And today, the agency continues to maintain that there is no health risk for children consuming perchlorate. This interpretation has become alarming given the agency’s own recent studies [30,17] that show an increase in perchlorate consumption by infants and toddlers since the chemical’s approval for use in dry food plastic packaging and food-handling equipment.

The case of perchlorate demonstrates the disconnect between the health risk posed by real-world exposure, especially for vulnerable populations with iodine deficiencies, and a regulatory system that appears to have stagnated when it comes to scientific principles and methods used in decision-making. There is an urgency to effectively close this gap between regulatory mandate and scientific practice. But it is not a problem that is easily solved. It will require a combination of statutory changes, a renewed commitment by the agency itself, and the difficult task of changing institutional culture and practices.

## Good science requires solid information and independent judgement

While the 1958 food law emphasized the need for testing chemicals and ensuring that they were safe before being allowed in food, the statute gives the FDA little authority to systematically collect chemical hazard and exposure information from businesses or to develop a postmarket monitoring program to keep track of uses of chemicals (that is, how much is used and in which foods). As a result, the agency has little information for evaluating safety or monitoring it over time—consider that a chemical approved for use in food in the 1960s may be used today in more foods and in packaging or processing without any additional review by the agency. The change in the levels used in food along with the advancements in science in the subsequent 50 years suggests that some review of the chemical’s safety is warranted. But without an ability to track uses of chemicals, the agency has no visibility into the volume or frequency of chemical use, nor does it regularly obtain new information on toxicity that could be used to prioritize re-evaluations of chemicals used in food.

Another statutory provision hindering the agency’s ability to know whether all the chemicals in the food supply are safe is the “generally recognized as safe” or GRAS loophole. [4] With this exemption, Congress intended that additives such as vinegar and oils should not undergo safety testing because of a long history of safe uses. In other words, food manufacturers could determine a substance’s use was GRAS without informing the FDA of its safety. Unfortunately, the FDA has interpreted this provision to mean that chemical and food manufacturers can declare any new substance or new use of a substance to be GRAS with no obligation to tell the agency about the identity of the substance, where it was used, how much of it was used, and if it was safe [31].

In a universe of more than 10,000 chemicals, at least 1,000 have completely avoided FDA scrutiny through the GRAS exemption [4]. In an effort to entice companies to inform the FDA about the safety of their GRAS chemicals, the agency created a voluntary program where manufacturers could send the safety assessment of a chemical use they determined was GRAS and ask for FDA review. The agency then offers a nonbinding opinion; in other words, the regulator becomes a peer reviewer rather than the decision-maker. This process effectively creates a conflict-laden safety assessment process by allowing an employee or consultant to a company that profits from a given chemical to make the decision about the safety of that chemical. This voluntary system is further problematic in that it allows a company not to submit the safety decision to the FDA for its nonbinding opinion as it leaves the agency entirely unaware of the presence of the chemical in the food supply and its safety [32]. Furthermore, under this voluntary system, a company may also withdraw a notice even after the FDA flags a concern, and any company could move ahead and put that questionable chemical on the market without any requirement to resubmit to the FDA for review. This process ties the hands of the agency entirely because it no longer has any authority to limit a chemical’s use if, in its review, the substance is found to raise safety concerns. The substance can still be marketed as GRAS, and no one—not competitors nor consumers—will know that there might be safety concerns.

By effectively delegating regulatory authority to the industry itself, the agency’s power and authority is significantly weakened. It might not then be surprising that the agency is slow to challenge its long-held scientific practices. Raising new scientific questions or demanding more information from the industry would presume a position of power and authority, as it risks putting the agency in conflict with the regulated industry.

Further, the agency’s lack of access to information on the toxicity and exposure of chemicals extends beyond GRAS substances. Consider the following example: for chemicals purposely added to food such as flavors, preservatives, and sweeteners, the FDA recommends the industry perform a month-long feeding study in laboratory animals. Yet less than 22% of almost 4,000 chemicals have sufficient data to estimate how much is safe to eat, and less than 7% were tested for developmental or reproductive effects [33]. The paucity of information is astonishing.

## Looking forward

It is too tempting to put all the blame at the foot of the FDA. Certainly, when it comes to managing the safety of chemicals in food, the FDA has been sluggish to modernize its science and is falling far short in effectively accounting for the safe use of thousands of chemicals in use today, including well-known hazardous substances like perchlorate. But there is an important factor to consider in evaluating the effectiveness of our regulatory process: resources, both human and financial. Current efforts to roll back and repeal health and safety standards and dramatically cut agency budgets is a central component of President Trump’s political agenda. Cutting the agency’s funding even further will not solve the problem of regulatory ineffectiveness but rather will amplify it, potentially further eroding the independence of the agency from the industry it regulates.

The FDA Office of Food Additives Safety—one of 12 offices in the Center for Food Safety and Applied Nutrition (CFSAN) [34]—responsible for regulating more than 10,000 chemicals and a multi-billion-dollar industry—has a little over 100 full-time technical staff. The fiscal year (FY) 2017 budget for CFSAN and related field activities (e.g., implementation of the Food Safety Modernization Act of 2011) in the Office of Regulatory Affairs is about US$1 billion [35]. This budget pales in comparison to the US$371 billion in packaged foods sales in 2015 [36].

Additional resources are a critical component of a multipronged approach needed to improve the FDA’s processes to ensure that chemicals in the food we eat are safe. If the FDA is to integrate new scientific understandings into its consideration of chemical safety, it needs access to adequate and up-to-date information; it needs to be able to incentivize the regulated entities to provide such information without statutory changes; and it needs to be able to conduct systematic safety reviews of priority chemicals that were approved decades ago and never since re-examined.

Today, more than ever, this country needs to reinvest in the scientific institutions that we all have long relied on to protect our health. The FDA’s efforts to manage the tens of thousands of chemicals in use in our food supply have fallen far short of the task at hand given the rapid changes in food technology and scientific research on chemicals. As the case of perchlorate shows, this failure is putting the health of our children at risk.

## Abbreviations

CFSAN: Center for Food Safety and Applied Nutrition
FAA: Food Additive Amendment
FDA: Food and Drug Administration
FFDCA: Federal Food, Drug and Cosmetics Act
FY: fiscal year
GRAS: generally recognized as safe
IQ: intelligence quotient
